# Variation in ω-3 and ω-6 Polyunsaturated Fatty Acids Produced by Different Phytoplankton Taxa at Early and Late Growth Phase

**DOI:** 10.3390/biom10040559

**Published:** 2020-04-06

**Authors:** Sami Taipale, Elina Peltomaa, Pauliina Salmi

**Affiliations:** 1 Department of Biological and Environmental Science, Nanoscience center, University of Jyväskylä, P.O. Box 35 (YA), 40014 Jyväskylä, Finland; 2 Institute of Atmospheric and Earth System Research (INAR)/Forest Sciences, University of Helsinki, P.O. Box 64, 00014 Helsinki, Finland; elina.peltomaa@helsinki.fi; 3 Helsinki Institute of Sustainability Science (HELSUS), University of Helsinki, P.O. Box 4 (Yliopistonkatu 3), 00014 Helsinki, Finland; 4 Faculty of Information Technology, University of Jyväskylä, P.O. Box 35, FI-40014 Jyväskylän, Finland; pauliina.u.m.salmi@jyu.fi

**Keywords:** polyunsaturated fatty acids, phytoplankton, freshwater, nutritional value

## Abstract

Phytoplankton synthesizes essential ω-3 and ω-6 polyunsaturated fatty acids (PUFA) for consumers in the aquatic food webs. Only certain phytoplankton taxa can synthesize eicosapentaenoic (EPA; 20:5ω3) and docosahexaenoic acid (DHA; 22:6ω3), whereas all phytoplankton taxa can synthesize shorter-chain ω-3 and ω-6 PUFA. Here, we experimentally studied how the proportion, concentration (per DW and cell-specific), and production (µg FA L^−1^ day^−1^) of ω-3 and ω-6 PUFA varied among six different phytoplankton main groups (16 freshwater strains) and between exponential and stationary growth phase. EPA and DHA concentrations, as dry weight, were similar among cryptophytes and diatoms. However, *Cryptomonas erosa* had two–27 times higher EPA and DHA content per cell than the other tested cryptophytes, diatoms, or golden algae. The growth was fastest with diatoms, green algae, and cyanobacteria, resulting in high production of medium chain ω-3 and ω-6 PUFA. Even though the dinoflagellate *Peridinium cinctum* grew slowly, the content of EPA and DHA per cell was high, resulting in a three- and 40-times higher production rate of EPA and DHA than in cryptophytes or diatoms. However, the production of EPA and DHA was 40 and three times higher in cryptophytes and diatoms than in golden algae (chrysophytes and synyrophytes), respectively. Our results show that phytoplankton taxon explains 56–84% and growth phase explains ~1% of variation in the cell-specific concentration and production of ω-3 and ω-6 PUFA, supporting understanding that certain phytoplankton taxa play major roles in the synthesis of essential fatty acids. Based on the average proportion of PUFA of dry weight during growth, we extrapolated the seasonal availability of PUFA during phytoplankton succession in a clear water lake. This extrapolation demonstrated notable seasonal and interannual variation, the availability of EPA and DHA being prominent in early and late summer, when dinoflagellates or diatoms increased.

## 1. Introduction

Phytoplankton, the microscopic primary producers, are central transformers and cyclers of energy and biomolecules in aquatic food webs [1]. The ability of phytoplankton to synthesize different biomolecules influences their nutritional values and reflects their productivity throughout the aquatic food web [2,3,4]. Among all biomolecules synthesized by phytoplankton, alfa-linolenic acid (ALA; 18:3ω3) and linoleic acid (LIN, 18:2ω6) can be considered as essential polyunsaturated fatty acids (PUFAs) since consumers cannot synthesize these de novo [5]. These medium-chain ω-3 and ω-6 PUFA are precursors for eicosapentaenoic acid (EPA, 20:5ω-3), docosahexanoic acid (DHA, 22:6ω-3), and arachidonic acid (ARA, 20:4w6), but due to the consumers’ limited ability to bioconvert them from ALA or LIN, they can be considered as physiologically essential [5,6]. The physiological importance of long-chain ω-3 and ω-6 PUFA varies by consumers. Usually, DHA appears to be the most retained FA for copepods and many fish, whereas EPA is the most retained FA for *Daphnia* and some benthic invertebrates [6,7,8,9,10]. However, *Daphnia* can grow and reproduce without EPA, whereas total ω-6 availability may negatively affect somatic growth of *Daphnia* [11]. The egg production and hatching success of marine copepods from the genus *Acartia* have been reported to be highly positively correlated with ALA, EPA, and DHA and negatively correlated with SDA and LIN [12,13]. More precisely, ALA had less effect on egg production and hatching success than EPA and DHA, and DHA had higher effect than EPA [13]. Nevertheless, EPA and DHA are not the only important PUFA for zooplankton, and thus, production of medium chain ω-3 and ω-6 PUFA can promote consumers’ optimal health.

Although phytoplankton can synthesize many different biomolecules (e.g., amino acids, sterols, carotenoids) [11], species containing high amounts of EPA and DHA are considered high-quality food for zooplankton [2,14]. Among freshwater phytoplankton, cryptophytes, dinoflagellates, golden algae, diatoms, and raphidophytes have been identified as EPA-synthesizing taxa and cryptophytes, dinoflagellates, golden algae, and euglenoids as DHA-synthesizing taxa [15,16,17]. In addition, some marine green algae and eustigmatophytes can synthesize EPA, and cryptophytes synthesize DHA [12]. Even though cyanobacteria and freshwater green algae cannot synthesize EPA or DHA, some cyanobacteria strains and all green algae can synthesize ALA and stearidonic acid (SDA, 18:4ω3) [16,18] and can contribute much or all their FA. In addition to long-chain and medium-chain PUFA, green algae and diatoms can synthesize 16 PUFA, which does not have physiological importance for aquatic consumers [19].

There is a gap in the knowledge on how efficient different phytoplankton groups are in producing different PUFAs and on how much PUFA content per cell varies among phytoplankton species and within phytoplankton groups. Current knowledge of production efficiency comes from biofuel studies and other applications and majorly focuses on fast growing taxa, e.g., non-EPA- and DHA-producing green algae, or in the optimization of PUFA production of specific species in certain growth conditions, utilizing, for example, industrial side streams [20,21]. These results are therefore not directly applicable when implemented to phytoplankton field data. Studies on laboratory cultures have shed light on the effects of environmental conditions on different phytoplankton taxa to synthesize PUFA [22]. The nutritional value of phytoplankton has shown to be dependent on growth rate regulated by ambient temperature and irradiance [23,24] or on nutrient stress experienced by the phytoplankton. Mitchell et al. [25] reported three–four times higher importance of phytoplankton taxa in relation to environmental conditions on PUFA contributions. However, they were not able to define how much the PUFA content (per biomass or cell) varied within phytoplankton groups or by environmental conditions. Taipale et al. [26] studied the nutritional values of natural phytoplankton communities in 107 boreal lakes sampled once for two summers. They found a negative pattern along nutrient concentration and nutritional value of phytoplankton; however, the variation in the predictability was rather high, suggesting that there are other factors influencing phytoplankton PUFA content.

The main aim of the current research was to study the connections between phytoplankton taxa and the production of ω-3 and ω-6 PUFA along their growth. Furthermore, we wanted to study how the nutritional value of phytoplankton changes when ω-3 and ω-6 PUFA content is calculated per cell instead of per biomass. For this experimental study, we cultured 16 strains from six phytoplankton main groups isolated from boreal and temperate freshwaters. We also studied how the abundance of certain phytoplankton groups influences the production of ω-3 and ω-6 PUFA in eutrophic lake by a calculation of PUFA concentrations based on phytoplankton biomasses. We hypothesized that strains belonging to cryptophytes, dinoflagellates, chrysophytes, and diatoms display higher concentrations—both proportion and cell specific—of ω-3 and ω-6 PUFAs than green algae and cyanobacteria both in early and late growth phases. Additionally, we hypothesized that production rates of the former algae group were higher than that of the latter.

## 2. Materials and Methods

### 2.1. Phytoplankton Culturing

To study how phytoplankton taxa and growth influence the contribution, content, and production of ω-3 and ω-6 PUFAs, we cultured 16 freshwater phytoplankton strains belonging to six phytoplankton main groups (Table 1). From now on, we refer to the strains by their main groups or genus for readability. Each phytoplankton strain was pre-cultured using MWC medium [27,28] with AF6 vitamins [29] at a temperature of 18 ± 1 °C, under 14 h:10 h light:dark cycle with a light intensity of 50–70 µmol m^−2^ s^−1^. For the actual experiment, we used 200 mL tissue tubes with 75 mL inoculum of pre-cultured algae and 125 mL of fresh MWC with AF6 vitamins. Each strain was cultured in three replicates. Cell density of phytoplankton cultures were measured prior and during the experiment by using an electronic cell counter (Casy, Omni Life Science, Bremen, Germany) with 60 µm capillary (measurement range 1.2–40 µm). Samples for fatty acid analyses were harvested by filtering 20–100 mL of phytoplankton culture onto cellulose nitrate membrane filters (pore size 3 µm, Whatman, Maidstone, Kent, UK).

The specific rates of increase (*r*_n_, divisions day^−1^) for all strains were calculated for the exponential growth phase using Equation (1):

*r*_n_ = ln(*N_t_*/*N*_0_)/t (1)

where *N*_0_ is a population at the beginning of the experiment, *N_t_* is the population size at the time *t* that was determined as the exponential growth phase at the time when the first fatty acid samples were harvested.

### 2.2. Lipid Extraction and Fatty Acid Methylation

Lipids were extracted from the filters using a chloroform:methanol 2:1 mixture and then sonicated for 10 min, after which 0.75 mL of distilled water was added. Samples were mixed by vortexing and centrifuged (2000 rpm) in Kimax glass tubes, after which the lower phase was transferred to a new Kimax tube. The solvent was evaporated to dryness. Fatty acids of total fraction were methylated using acidic conditions. Toluene and sulfuric acid were used for the transesterification of fatty acid methyl esters (FAMEs) at 50 °C for 16 h, which is the optimal method for methylation PUFA [30]. FAMEs were analyzed with a gas chromatograph (Shimadzu Ultra, Kyoto, Japan) equipped with mass detector (GC-MS) and using helium as a carrier gas (linear velocity = 36.3 cm s^−1^). The temperature of the injector was 270 °C and we used a splitless injection mode (for 1 min). Temperatures of the interface and ion source were 250 °C and 220 °C, respectively. Phenomenex^®^ (Torrance, CA, USA) ZB-FAME column (30 m × 0.25 mm × 0.20 µm) with 5 m Guardian was used with the following temperature program: 50 °C was maintained for 1 min, then the temperature was increased at 10 °C min^−1^ to 130 °C, followed by 7 °C min^−1^ to 180 °C, and 2 °C min^−1^ to 200 °C. This temperature was held for 3 min, and finally, the temperature increased 10 °C min^−1^ to 260 °C. The total program time was 35.14 minutes and solvent cut time was 9 minutes. Fatty acids were identified by the retention times (RT) and using specific ions [18], which were also used for quantification. Fatty acid concentrations were calculated using calibration curves based on known standard solutions (15 ng, 50 ng, 100 ng and 250 ng) of a FAME standard mixture (GLC standard mixture 566c, Nu-Chek Prep, Elysian, MI, USA) and using recovery percentage of internal standards. The Pearson correlation coefficient was >0.99 for each individual fatty acid calibration curve. Additionally, we used 1,2-dinonadecanoyl-sn-glycero-3- phosphatidylcholine (Larodan, Malmö, Sweden) and free fatty acid of C_23:0_ (Larodan, Malmö, Sweden) as internal standards and for the calculation of the recovery percentages.

### 2.3. Quantitation of Fatty Acids

Here, we focused on two medium chain ω-3 (ALA, SDA) and two ω-6 (LIN, GLA) PUFA and two long-chain ω-3 (EPA, DHA) and ω-6 (ARA, DPA) PUFA. However, we calculated the contribution of these PUFA from all quantified fatty acids. In addition to the contribution of PUFA, we calculated their content per phytoplankton dry weight biomass and per cell. The fatty acid content (µg in mg) was calculated based on the following Equation (2):

(2) $$\frac{Q_{FA}\times V_{vial}}{DW_{1}\times R_{p}}$$

where *Q_FA_* is the concentration of the fatty acid (µg µL^−1^) based on calibration curves of GLC-566C (Nu-Chek Prep, Elysian, MN, USA) for each fatty acid, *V_vial_* denotes the running volume of the samples (µL), *DW*_1_ is dry weight of the sample, and *R_p_* denotes the recovery percentage based on internal standards.

We calculated ω-3 and ω-6 PUFA content per phytoplankton carbon biomass. The fatty acid content (µg in mg C) was calculated based on Equation (3):

(3) $$\frac{Q_{FA}\times V_{vial}}{V_{filtered}\times TCBM\times R_{p}}$$

where *Q_FA_* is the concentration of the fatty acid (µg µL^−1^), *V_vial_* denotes the running volume of the samples (µL), *V_filtered_* is the total volume of filtered lake water (L), *TCBM* denotes the total phytoplankton carbon biomass (μg C L^−1^) of the corresponding sample, and *R_p_* denotes the recovery percentage based on internal standards.

The cell-specific fatty acid concentration (pg in cell) was calculated based on Equation (4):

(4) $$\frac{Q_{FA}\times V_{vial}}{V_{filtered}\times Cell\times R_{p}}$$

where *Q_FA_* is the concentration of the fatty acid (µg µL^−1^), *V_vial_* denotes the running volume of the samples (µL), *V_filtered_* is the total volume of filtered of cultured phytoplankton (L), *Cell* is the number of cells of the culture, and *R_p_* denotes the recovery percentage based on internal standards.

Additionally, daily production of PUFA (µg L^−1^ Day^−1^) was calculated based on Equation (5):

(5) $$\frac{Q_{FA}*V_{vial}}{DW_{1}*R_{p}}\times \frac{DW_{2}/V_{filtered}}{Days}$$

where *Q_FA_* is the concentration of the fatty acid (µg µL^−1^), *V_vial_* denotes the running volume of the samples (µL), *DW*_1_ is dry weight of the sample, and *R_p_* denotes the recovery percentage based on internal standards. *DW*_2_ is dry weight of the phytoplankton samples between time 1 (e.g., initial) and 2 (e.g., exponential phase), *V_filtered_* is the total volume of filtered of cultured phytoplankton (L), and Days cites to the number of culturing days between time 1 and 2.

### 2.4. Statistical Methods

Bray Curtis similarity matrix of fatty acid data was created using Primer 7^81^ (Plymouth Routines In Multivariate Ecological Research, Primer E) of which a non-metric multidimensional scaling (NMDS) plot was created. CLUSTER analysis (Hierarchical Cluster analysis) was used to create 70% similarities in the NMDS ordination. PERMANOVA (Permutational multivariate analysis of variance [31]) was used to test if differences in the ω-3 and ω-6 PUFA composition, biomass, and cell content and production were statistically significant between phytoplantkon groups and growth phase. PERMANOVA was run with unrestricted permutation of raw data and type III sums of squares. Similarity percentages (SIMPER) were used to detect how different units influence the similarity within phytoplankton group and to identify the characteristic fatty acids of each phytoplankton group. We used PERMDISP (Distance-based test for homogeneity of multivariate dispersions [32]) to investigate the within-class variation in ω-3 and ω-6 PUFA composition, biomass, and cell content and production.

### 2.5. Implementing Laboratory Culturing Data on Field Data

To scrutinize the phenology of PUFA availability in a well-studied urban lake, phytoplankton data from the Enonselkä basin of Lake Vesijärvi, Central Finland (WGS84 61°2.2′N, 25°31.7′E), were taken from the Hertta database of the Finnish Environment Institute (requires registration, https://www.syke.fi/avointieto). Phytoplankton countings saved in the database were done using accredited method (EN 16695, 2015) by the Finnish Environment Institute. Lake Vesijärvi is a eutrophic, clear water lake (total phosphorus 27 µg L^−1^ and water color 10 mg Pt L^−1^, Finnish Environment Institute, Water Framework Directive classification and status assessment) regularly experiencing blooms of cyanobacteria and diatoms.

Phytoplankton biomasses (mg C L^−1^) from open water seasons 2015–2018 (five–six samplings in May–November), including contrasting cyanobacteria-dominant years and years without cyanobacteria blooms, were used to form comparisons with the experimental design. For this, the counted phytoplankton taxa were divided into main taxa: cryptophytes, cyanobacteria, diatoms, dinoflagellates, golden algae, and green algae that included also conjugatophytes. Other reported algae were classified as “other.” Phytoplankton biomasses were converted to PUFA availabilities by using the amount of each compound in the experimental study as an average dry weight per mg in exponential and stationary phase. A coefficient of 0.45 was used to convert dry weight to carbon biomass based on our previous measurements [33]. If the experimentally studied main taxon included several tested strains, such as cryptophytes, included the *Cryptomonas* and *Rhodomonas* species, the average of the two strains was used. This was based on the analysis of experimental data, illustrating that the main taxa explained most of the variation in the fatty acid composition as µg FA per mg dry weight.

## 3. Results

### 3.1. Growth Rate

**Figure 1:**
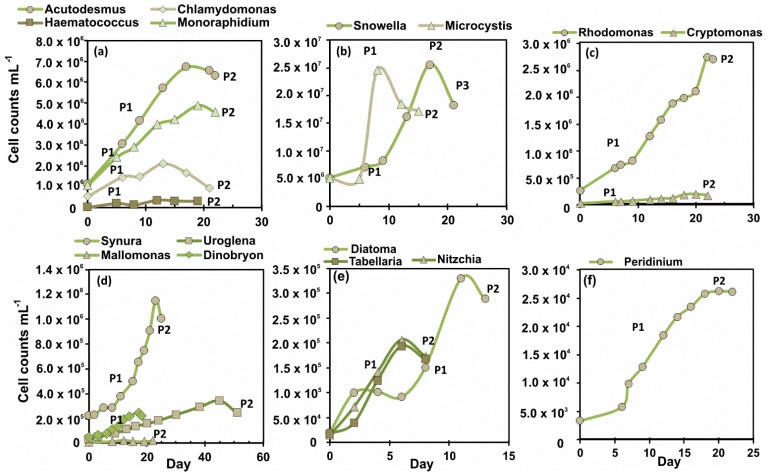
Growth curves for 16 cultures of phytoplankton strains classified by phytoplankton groups: (**a**) green algae, (**b**) cyanobacteria, (**c**) cryptophytes, (**d**) golden algae including chrysophytes and synyrophytes, (**e**) diatoms, and (**f**) dinoflagellate. P1 cites to sampling point during exponential growth phase and P2 cites to the sampling point in stationary phase.

Cell abundance was highest (2.5 × 10^7^) with cultured cyanobacteria strains but remained low (<2.5 × 10^4^ cell mL^−1^) throughout 22 days in cultures of *Mallomonas*. Growth rate (Table 1, Figure 1) between initial and the middle of exponential growth phase was highest with all three strains of diatoms (*Nitzchia*, *Tabellaria* and *Diatoma*) and second-highest with *Haematococcus* (green algae; 0.38 divisions d^−1^) and *Microcystis* (cyanobacteria; 0.21 divisions d^−1^), even though *Haematococcus* culture did not reach high density. Growth rates were slowest with strains of golden algae of *Synura*, *Mallomonas,* and *Uroglena*, and then with dinoflagellate *Peridinium*. Diatoms reached stationary phase already in eight–13 days, whereas it took 51 days for *Uroglena* to reach the stationary phase.

### 3.2. Phytoplankton Taxa and Growth Phase Impact on the Contribution of ω-3 and ω-6 PUFA

**Figure 2:**
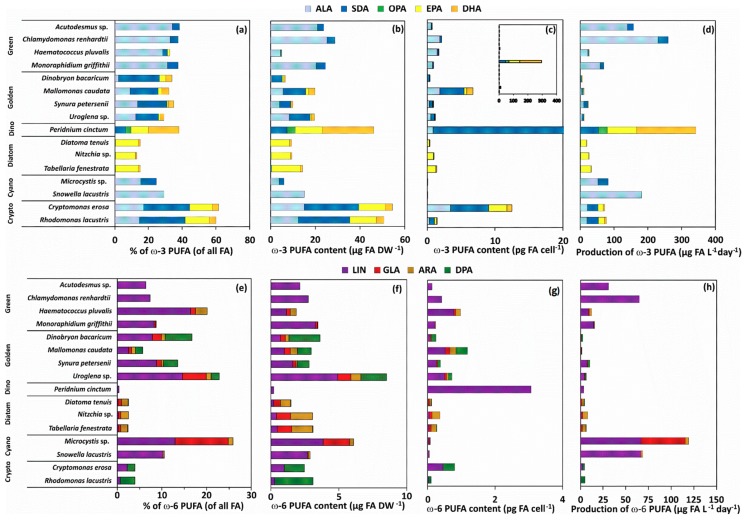
The proportion of all fatty acids (**a**,**e**), per biomass content (**b**,**f**), per cell content (**c**,**g**), and daily production (**d**,**h**) of ω-3 (ALA, SDA, OPA, EPA, DHA) and ω-6 (LIN, GLA, ARA, DPA) PUFA in cultured 16 phytoplankton strains.

**Figure 3:**
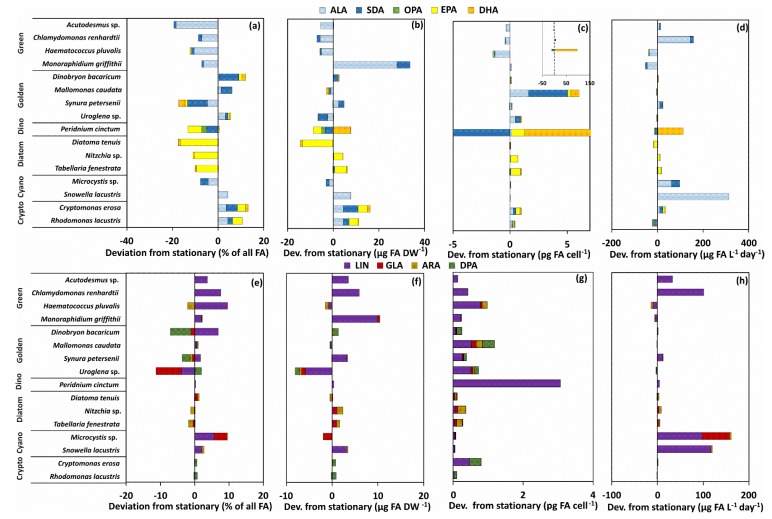
The deviation of ω-3 (ALA, SDA, OPA, EPA, DHA) and ω-6 (LIN, GLA, ARA, DPA) PUFA between exponential and stationary phases: proportion of all fatty acids (**a**,**e**), per biomass content (**b**,**f**), per cell content (**c**,**g**), and daily production (**d**,**h**).

The contribution of ω-3 and ω-6 PUFA of 16 phytoplankton strains varied by the phytoplankton group (Figure 2), but also by growth phase (Figure 3). All strains of green algae and cyanobacteria contained ALA, SDA, and LIN, excluding *Snowella* that did not contain any SDA. The contribution of GLA was highest in *Microcystis*, whereas trace amounts were found among golden algae, diatoms, and green algae. In addition to medium-chain ω-3 and ω-6 PUFA, diatoms, golden algae, and the dinoflagellate contained also EPA and DHA. The absolute contribution of ALA was highest in green algae and *Snowella* (~30% of all FA), whereas cryptophytes and *Dinobryon* had the highest (~26% of all FA) contribution of SDA among all phytoplankton strains. Octadecapentaenoic acid (OPA, 18:5ω3) was found only from the dinoflagellate *Peridinium cinctum* (~4% of all FA). The contribution of LIN was highest (~10% of all FA) in *Haematococcus*, *Uroglena*, *Mallomonas*, and *Synura*, whereas diatoms and the dinoflagellate had only a minor contribution of LIN (<1% of all FA). All strains of cryptophytes, diatoms, and the dinoflagellate had equal contribution of EPA (~13% of all FA), whereas the contribution of DHA was highest (18.4 ± 0.2 % of all FA) in *Peridinium*. Additionally, cryptophytes and golden algae contained also docosapentaenoic acid (ω-6 DPA).

**Figure 4:**
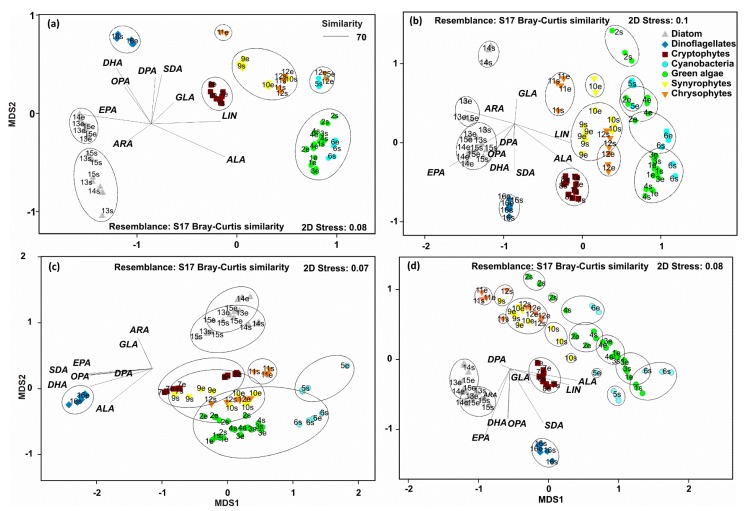
Non-metric multidimensional scaling plots of Bray Curtis similarity of percentages (**a**), biomass content (**b**), cell content (**c**), and daily production (**d**) of ω-3 (ALA, SDA, EPA, DHA) and ω-6 (LIN, GLA, ARA, DPA) PUFA in cultured 16 phytoplankton strains (see Table 1). Golden algae are divided here into Synyrophytes and Chrysophytes to demonstrate the difference in these classes. Abbreviations after strain number: e = exponential phase and s = stationary phase.

**Figure 5:**
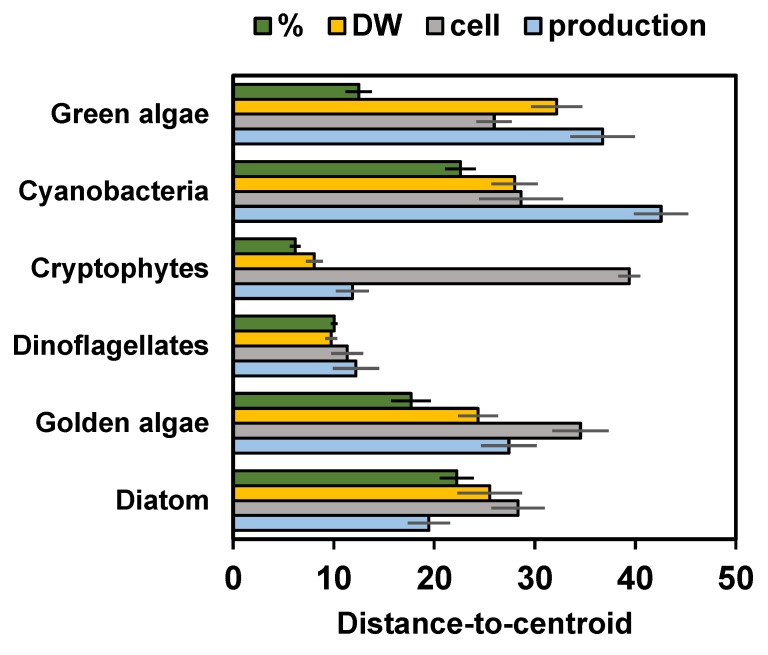
Permutational Analysis of Multivariate dispersion (PERMDISP) of ω-3 and ω-6 PUFA across each phytoplankton class (contribution (%), biomass content (DW), cell content (cell), and production.

According to the PERMANOVA (Table 2) the contribution of ω-3 and ω-6 PUFA differed between strains by the taxa, but also by the growth phase. Taxa explained 84% of all variation, but growth phase explained only 1% of the variation. Pairwise PERMANOVA (t = 2.58–27.8, P(MC) < 0.003) showed that the contribution of ω-3 and ω-6 PUFA differed among phytoplankton main groups. However, non-metric multidimensional scaling analysis (Figure 4) clustered (CLUSTER analysis) *Snowella* with green algae and *Microcystis* with exponential phase of *Uroglena* together by 70% similarity excluding. Furthermore, NMDS output of percentages of ω-3 and ω-6 PUFA separated strains by growth phase. Pairwise PERMANOVA (t = 3.7–7.1, P(MC) = 0.001) showed statistical difference between exponential and stationary phase for green algae, diatoms, dinoflagellates, and cryptophytes, but not for cyanobacteria or golden algae (t = 0.75–1.01, P(MC) = 0.35–0.55). The contribution of ω-3 PUFA was higher in exponential phase in green algae, dinoflagellates, and diatoms, whereas cryptophytes and chrysophytes (excluding *Synura*) had higher contribution of different ω-3 PUFA in stationary phase (Figure 3). The contribution of LIN in green algae and cyanobacteria was higher in stationary phase than in exponential phase. Otherwise, similar clear trends were not seen in the contribution of ω-6 PUFA with other taxa. Permutational analysis of multivariate dispersions (PERMDISP) showed lowest dispersion among cryptophytes and green algae, whereas dispersion was highest within cyanobacteria (Figure 5) reflecting high variation among these phytoplankton classes (Figure 3a).

### 3.3. Phytoplankton Taxa and Growth Phase Impact on the Content of ω-3 and ω-6 PUFA

The biomass (DW) and cell content of individual ω-3 and ω-6 PUFA varied greatly among 16 phytoplankton strains (Figure 2). According to the PERMANOVA (Table 2) the content (per biomass and cell) of ω-3 and ω-6 PUFA differed by the phytoplankton group, but not by the growth phase. Phytoplankton taxa explained 69% and 65% of all variation for biomass and cell contents, respectively. Pairwise PERMANOVA (t = 2.3–9.6, P(MC) = 0.001–0.008) comparison showed that all phytoplankton groups differed from each other when PUFA content was calculated per cell but not between cyanobacteria and green algae when PUFA content was calculated per biomass (t = 1.615, P(MC) = 0.071). Total biomass content of ω-3 PUFA was highest in cryptophytes (Figure 2), but when ω-3 PUFA content was calculated per cell the dinoflagellate *Peridinium* had 24-fold content of ω-3 PUFA in relation to any phytoplankton strain (Figure 2). More specifically, green algae excluding *Haematococcus* had highest ALA content per biomass, cryptophytes had the highest SDA content and cryptophytes, diatoms, and dinoflagellates had the highest EPA content. *Peridinium* had seven times higher DHA content than in any other phytoplankton strains. Total ω-6 PUFA biomass content was highest among *Uroglena* and *Microcystis*, which had both especially high LIN and GLA. Additionally, all cryptophytes and golden algae had relatively high ω-6 DPA content.

Dispersion (PERMDISP) of ω-3 and ω-6 PUFA per DW was low (Figure 5) and group similarity was high (SIMPER; Table 3) only among cryptophytes and dinoflagellates (including only one species of exponential and stationary). When using per cell PUFA concentrations in PERMDISP analysis, dispersion was high and similarity low among all phytoplankton. This trend was especially seen with golden algae and cryptophytes: cell ω-3 and ω-6 PUFA content was relatively higher in *Mallomonas* and *Cryptomonas* than in other species of golden algae or cryptophytes, respectively. The output of non-metric multidimensional scaling of ω-3 and ω-6 PUFA content (Figure 4b,c) also showed that dissimilarity within phytoplankton group is higher when PUFA content is calculated per cell than per biomass. This was especially seen between golden algae and cryptophytes that clustered separately in NMDS when using per biomass content but did not differ in NMDS when per cell content was used. We found logarithmic regression (y = 2.9093ln(x) + 8.0141; r^2^ = 0.645) between cell size and ω-3 PUFA content per cell. The per biomass content of ω-3 and ω-6 PUFA of phytoplankton strains in exponential and stationary phase varied greatly within phytoplankton groups, and cryptophytes were the only group in which both strains had higher PUFA content in stationary than in exponential phase. When the ω-3 PUFA content was calculated per cell, all cultured strains excluding *Acutodesmus, Chlamydomonas,* and *Haematococcus* had equal or higher ω-3 PUFA content per cell in stationary than in exponential phase (Figure 3).

### 3.4. Phytoplankton Taxa and Growth Phase Impact on the Production of ω-3 and ω-6 PUFA

The production of medium-chain and long-chain ω-3 and ω-6 PUFA differed (PERMANOVA, Table 2) according to phytoplankton class (Figure 2d,h), within the phytoplankton main group (PERMDISP and SIMPER; Figure 5, Table 3), and by the growth phase (Table 2). However, growth phase explained only 1% of the variation, whereas phytoplankton taxa explained 66% of all PUFA variation. Pairwise PERMANOVA (t = 4.80–10.37; P(MC) = 0.001) showed that all phytoplankton groups, excluding cyanobacteria and green algae, differed from each other (t = 1.39, P(MC) = 0.124). Production of ω-3 and ω-6 PUFA differed by growth phase among diatoms and cyanobacteria (Pairwise PERMANOVA: t = 1.93–3.38; P(MC) = 0.001–0.041). The production of ALA was highest with green algae (*Chlamydomonas*, *Acutodesmus*) and cyanobacteria (*Snowella*), whereas dinoflagellate (*Peridinium*) and cryptophytes had the highest production of SDA per day. The dinoflagellate *Peridinium* produced three and 33 times more EPA and DHA per day (µg PUFA L^−1^ day^−1^), respectively, than any other phytoplankton strain. Diatoms had highest production values for EPA and cryptophytes for DHA after *Peridinium*. Furthermore, diatoms and cryptophytes had 87 and 34 times higher production of EPA than chrysophytes, respectively. Production of LIN was highest in cyanobacteria and *Chlamydomonas* and *Acutodesmus*, whereas *Microcystis* alone had highest production of GLA. Cryptophytes and golden algae produced highest amount of ω-6 DPA in a day, even though it was relatively low in comparison with the production of LIN produced by green algae and cyanobacteria. Similarity analysis (SIMPER) showed that similarity in the production ω-3 and ω-6 PUFA was highest among cryptophytes and diatoms, whereas the similarity (SIMPER) was lowest with green algae and cyanobacteria. Green algae and cyanobacteria also clustered together in the NMDS plot. Production of ω-3 and ω-6 PUFA did not differ statistically between the exponential and stationary growth phase at the main group level, but some strains, e.g., *Chlamydomonas*, *Microcystis*, and *Snowella*, had a relatively higher production of ALA and LIN at the stationary phase (Figure 4d,h).

### 3.5. Extrapolation to Field Data

**Figure 6:**
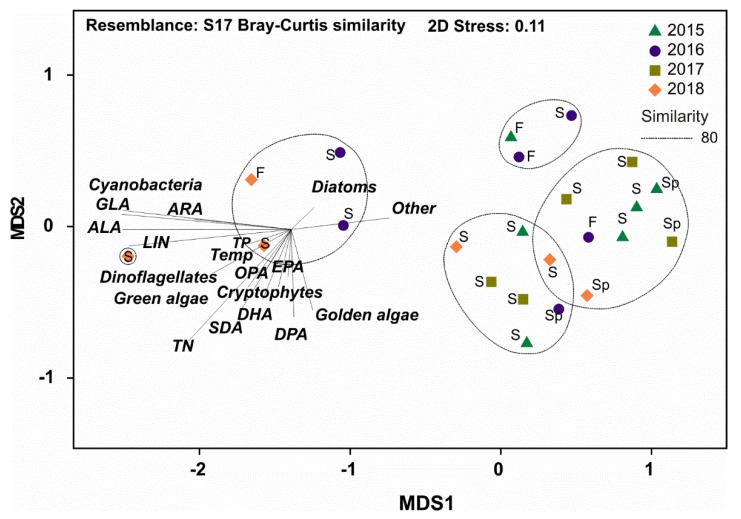
Non-metric multidimensional scaling plots of Bray Curtis similarity of ω-3 (ALA, SDA, EPA, DHA) and ω-6 (LIN, GLA, ARA, DPA) PUFA concentration of phytoplankton (µg PUFA L ^−1^), main phytoplankton groups and physico-chemical parameters in Lake Vesijärvi in years 2015–2018. TN—Total Nitrogen, TP—Total Phosphorus, Temp—temperature in the epilimnion. S = summer, F = fall, Sp = Spring.

The community composition in Lake Vesijärvi had no clear pattern during the study years (Figure 6, Figures S1 and S2). However, the proportion of dinoflagellates was generally highest in spring. In June 2015, cryptophytes and golden algae increased and were followed by diatoms and cyanobacteria in autumn. On the contrary, years 2016 and 2018 were dominated by cyanobacteria from June until autumn, whereas in 2017, cryptophytes and diatoms increased in mid-summer and cyanobacteria in autumn.

Converted to fatty-acid availabilities, the concentration of ω-3 and ω-6 PUFA did not differ between years (PERMANOVA: Pseudo-F = 1.49, *p* = 0.195), but field data demonstrated notable seasonal and interannual variation (PERMANOVA: Pseudo-F = 4.36, *p* = 0.007). According to the two factor PERMANOVA, the season explained 24% of all variation in the PUFA concentrations. Generally, non-metric multidimensional scaling clustered phytoplankton and corresponding PUFA concentrations in four groups with 80% similarity (Figure 6). NMDS1 correlated strongly negatively (*r* = −0.98) with cyanobacteria. One point was close with cyanobacteria, and four points related closely with diatoms and all other sampling points were in the right side of the NMDS output. Different PUFA showed a strong relationship with certain phytoplankton groups. Cyanobacteria-dominance was reflected as the high concentration and proportion of ALA, LIN, and GLA throughout the growing season (Pearson correlation: *r* = 0.93–0.97, *p* < 0.001), which peaked after midsummer. The relative proportion of DHA was highest in early summer, when biomass of dinoflagellates was relatively high (Figure 6, Figure S1). The concentration of DHA showed a strong correlation with the biomass of dinoflagellates (Pearson correlation: *r* = 0.957, *p* < 0.001), whereas the concentration of EPA was most closely related with diatoms (Pearson correlation: *r* = 0.648, *p* < 0.001) and cryptophytes (Pearson correlation: *r* = 0.671, *p* < 0.001). However, NMDS output separated diatoms as their own group, and EPA was more closely related with golden algae and cryptophytes than with diatoms. The abundance of green algae showed strong correlation with biomass of cryptophytes and dinoflagellates (Pearson correlation: *r* = 0.47–0.59, *p* < 0.004–0.022), as can be seen in the NMDS output (Figure 6), resulting in a strong inter-correlation with the concentration of EPA and DHA (Pearson correlation: *r* = 0.57–0.72, *p* < 0.0001–0.005). Whereas total phosphorus (TP) was positively related with cyanobacteria in NMDS output, temperature was positively related with dinoflagellates, cryptophytes, golden algae, and green algae. However, a negative relationship between cyanobacteria and TP was not statistically significant (*r* = 0.406, *p* = 0.055).

## 4. Discussion

The experimental setup of this study consisted of six main groups of phytoplankton (cryptophytes, dinoflagellates, golden algae, diatoms, green algae, and cyanobacteria), which were sampled at early and late growth phase to understand how phytoplankton nutritional value and production of ω-3 and ω-6 PUFA may vary along phytoplankton growth. Inclusion of one–four different strains in each main group facilitated scrutinization of variation inside taxonomic main groups. Briefly, even though the ability to synthesize different ω-3 and ω-6 PUFA follows strictly phylogenetical groups [15,16,22,34], the PUFA content per cell and the production of PUFA can vary greatly within phytoplankton groups.

Typically, the studies on phytoplankton fatty acids report the contribution of different PUFA together with the total concentrations of PUFA (e.g., per dry weight or carbon) [15,16]. Deviating from the previous studies, we determined the cell-specific fatty acid content and production rates for the main freshwater phytoplankton groups. Proportions, dry weights, and cell-specific concentrations were calculated for both exponential and stationary growth phase. Our results revealed that cell-specific PUFA content differed greatly from biomass-specific PUFA content and the variation in cell PUFA content within phytoplankton group was high likely due to the variable size of phytoplankton. Comparison of the different metrics demonstrated risk of being misled if scrutinizing only one type of concentration and making ecological extrapolation. Proportion and concentration as dry weight can give only restricted amount of information on PUFA and might be of more interest in biofuel production [19]. However, information of the cell-specificity is important, because in plankton communities, secondary consumers feed on a diverse phytoplankton community, and the size of the animal is proportional to the size of the phytoplankton that it can ingest [1,15].

In this study, *Peridium* had a large cell diameter and relatively slow specific growth rate, both characteristics typical of *K*-strategists displaying resource-efficiency in traditional r/K classification [1]. In Lake Vesijärvi, dinoflagellates occurred at the time typical for cells displaying these functional traits. However, DHA content per DW was seven times higher in *Peridium* than in any other phytoplankton strain, whereas DHA content per cell in *Peridium* was ~200 fold in relation to any other phytoplankton strain. This makes a many-fold difference for filter-feeding zooplankton grazers, and explains why dinoflagellates are the preferable diet for copepods [35]. *Daphnia* do not grow well with *Peridinium*, maybe due to the armoring and low amounts of sterols [11]. However, according to the fatty acid modeling, *Daphnia*’s diet consisted of ~20% dinoflagellates in Lake Vesijärvi in year 2016 [26]. Therefore, it seems that dinoflagellates can fuel EPA and DHA demand of both zooplankton groups and the whole food web as seen earlier in a strong correlation between the biomass of dinoflagellates and DHA content of perch [17]. Even though *Peridinium* grew slowly, we found that the production of DHA was 40 times higher with *Peridinium* than any other phytoplankton strain, which emphasizes the role of this non-toxic freshwater dinoflagellate in the synthesis of DHA. Therefore, even a small increase in the biomass of *Peridinium* can significantly increase the production of DHA in boreal lakes. However, some dinoflagellates species, e.g., *Ceratium*, are too large for zooplankton to ingest, and thus, high DHA content in them is not available for zooplankton.

Herbivorous cladoceran can have a high proportion of EPA, whereas DHA is nearly absent in them [10,36,37]. Therefore, the production of EPA is important for herbivorous cladocerans (e.g., *Daphnia*). Diatoms and cryptophytes are crucial producers of EPA in freshwaters [38]. Meanwhile, the percentage and biomass content of EPA is similar with cryptophytes and diatoms. Our results showed that cell content of EPA varies greatly between these two phytoplankton groups. Meanwhile, *Cryptomonas* had a higher EPA content per cell than any of the studied diatoms. We found the lowest cell EPA content in *Rhodomonas*. Moreover, since diatoms grow faster than cryptophytes, we found 2.3 times higher production of EPA with diatoms than with cryptophytes. These two phytoplankton groups equally influenced the concentration of EPA in Lake Vesijärvi, showing the importance of diatoms, especially in spring and autumn, while cryptophytes’ importance was largely shown in summer. Furthermore, our previous fatty acid-based modeling on the composition of *Daphnia* diets also showed that cryptophytes and diatoms are the two main dietary sources of this key herbivorous zooplankton in Lake Vesijärvi [26]. However, the size and form of diatoms vary greatly, and they have silica frustules that might be difficult for *Daphnia* to ingest. Therefore, digestibility of diatoms varies greatly. Furthermore, diatoms can form large colonies and blooms, which are not ingestible for zooplankton, resulting in poor utilization of the diatom-produced EPA. Moreover, previous studies have shown that the EPA content of different species and by habitat is highly variable [13,39,40].

Even though ω-3 and ω-6 PUFA profiles of cyanobacteria and green algae differ at some level, the biomass and cell content of these PUFA did not differ markedly but were clustered together in NMDS output. This results from the fact that both phytoplankton groups grow fast and have a high ALA and LIN content. Our results also showed that these two groups were superior in producing ALA and LIN, which is one reason why they have been used for biofuel production. However, in terms of efficient transfer of these medium-chain PUFA in aquatic food webs, phytoplankton need to be digestible for zooplankton, and zooplankton need to have the ability to bioconvert EPA or DHA from ALA or ARA from LIN. Generally, it has been assumed that zooplankton does not feed on especially large-sized cyanobacteria, whereas other studies suggest that zooplankton can feed on cyanobacteria [34,35]. In Lake Vesijärvi, cyanobacteria (e.g., *Planktothrix*, *Snowella*, *Aphanizomenon*, *Microcystis*) can form blooms that can last throughout summer, as were seen in 2016. According to the fatty acid-based modeling [26], cyanobacteria formed less than 10% of the diet of *Daphnia,* and when the model uncertainties were considered, it could be noted that cyanobacteria were an insignificant diet source for *Daphnia*. Therefore, it seems that cyanobacteria may contain much of ALA and LIN but remain an inaccessible resource for zooplankton. Secondly, it should be noted that *Daphnia* has a poor ability to bioconvert EPA from ALA [41,42].

Here, we focused on phytoplankton phylogeny and growth phase and were unable to extrapolate the environmental conditions’ impact on production of ω-3 and ω-6 PUFA, since we converted phytoplankton biomass to fatty-acid availabilities in our field data. However, our field data showed a positive relationship between total phosphorus and ALA, LIN, and GLA production by cyanobacteria, whereas increased temperature and total nitrogen was related with the production of SDA, EPA, DHA, and ω-6 DPA by cryptophytes, golden algae, and dinoflagellates. In addition to changes in phytoplankton composition, environmental conditions can affect phytoplankton PUFA content [43,44,45], and thus, potentially, also their production. Our recent study [46] with 107 boreal lakes showed that intensified eutrophication decreases the nutritional value of phytoplankton. The high difference in temperature between freshwater and brackish and marine phytoplankton strains resulted in a 10-fold difference in the production of EPA [39]. Another study [43] with green algae, cryptophytes, and diatoms showed that the light and temperature increase (from 10 to 25 °C) have a relatively minor impact on PUFA content in green algae. Surprisingly, in our study, slow growing cryptophytes and golden algae had higher EPA contribution in stationary phase, whereas fast growing diatoms and slower growing dinoflagellates and synurophytes had higher EPA contribution in the exponential than in the stationary phase. The same trend was also seen in the biomass and cell PUFA content of cryptophytes, dinoflagellate, and diatoms, excluding *Diatoma*, which had minimal PUFA content in the stationary phase. However, the effect of the growth phase on the EPA production of cryptophytes, golden algae, dinoflagellate, and diatoms was ambiguous, showing that the production of EPA can vary within phytoplankton groups. The growth phase had a small impact on the *Peridinium* biomass and cell EPA content, but *Peridinium* had two times higher DHA content per cell and production of DHA in stationary than in exponential phase. The contribution of ALA and SDA of green algae was higher in stationary than in exponential phase; however, the biomass and cell content and the production of ALA and SDA varied greatly by green algae strains. The growth phase affected the contribution, content, and production of ALA and SDA differently. Altogether, it seemed that the growth phase together with the environmental parameters could affect PUFA content and production of freshwater phytoplankton.

Based on their capability to overcome and adapt to environmental constrains, phytoplankton can be categorized into functional groups [44,45,47]. Functional classification may include growth and morphometric traits that determine how easily a phytoplankter is eaten by a consumer [47]. This could be an important approach, because it includes both environmental conditions and phytoplankton physiological traits, and modern food web models typically use functional rather than phylogenetic phytoplankton inputs [48]. Here, we focused on growth rate and cell size; however, future studies might benefit from using phytoplankton strains from different functional groups.

## 5. Conclusions

In conclusion, for understanding the synthesis and transfer of ω-3 and ω-6 PUFA, calculations of PUFA content per phytoplankton cell are beneficial in addition to biomass content. Our results showed that phytoplankton PUFA per biomass content varies from the cell PUFA content due to the positive impact of cell size on PUFA content. Therefore, larger cells have a higher PUFA content than smaller cells, but too large cells are not digestible for herbivorous zooplankton, and subsequently, are not utilized or transferred in the freshwater food web. Our laboratory culturing emphasized that different ω-3 and ω-6 PUFA are synthesized by certain phytoplankton taxa. Extrapolation on field phytoplankton data demonstrated how the availability of PUFA differed inter- and intra-annually. Dinoflagellates were superior producers of DHA, whereas diatoms and cryptophytes were crucial producers of EPA in boreal lakes. Our results also demonstrated that phytoplankton PUFA content and production varied by growth phase; however, this change is difficult to predict due to the high variation between strains within the same phytoplankton groups.

## Figures and Tables

**Table 1:** Cultured phytoplankton strains (taxa, order, species, and strain number), their mean size (diameter by electronic cell counter, µm), and growth phase rate (divisions d^−1^) for the exponential phase (P1, sampling point 1) and stationary phase (P2, sampling point 2)

Taxa	Order	Species	Strain	Nr.	Size (µm)	Growth P1	Growth P2
Chlorophyceae (green algae)	Chlamydomonadales	*Chlamydomonas reinhardtii*	NIVA K-1016	1	6.1	0.14 ± 0.00	−0.02 ± 0.01
	Chlamydomonadales	*Haematococcus pluvialis*	NIVA K-0084	2	17	0.38 ± 0.10	0.07 ± 0.03
	Sphaeropleales	*Acutodesmus* sp.	University of Basel	3	5	0.11 ± 0.00	−0.12 ± 0.00
	Sphaeropleales	*Monoraphidium griffithii*	NIVA-CHL 8	4	4.6	0.11 ± 0.04	0.07 ± 0.03
Cyanophyceae (cyanobacteria)	Chroococcales	*Microcystis* sp.	NIVA-CYA 642	5	4.1	0.21 ± 0.01	−0.06 ± 0.02
	Synechococcales	*Snowella lacustris*	NIVA-CYA 339	6	2	0.05 ± 0.00	−0.08 ± 0.00
Cryptophyceae (cryptophytes)	Cryptomonadales	*Cryptomonas erosa*	CPCC 446	7	6.14	0.09 ± 0.04	0.06 ± 0.00
	Pyrenomonadales	*Rhodomonas lacustris*	NIVA 8/82	8	11.04	0.12 ± 0.03	0.08 ± 0.00
Synyrophyceae (golden algae)	Synurales	*Mallomonas caudata*	CCAP 929/8	9	12.5	0.05 ± 0.00	−0.08 ± 0.00
	Synurales	*Synura petersenii*	CCAP 960/3	10	8.8	0.05 ± 0.00	0.07 ± 0.01
Chrysophyceae (golden algae)		*Dinobryon bavaricum*	CCAC 2950B	11	5.6	0.12 ± 0.02	0.09 ± 0.00
	Chromulinales	*Uroglena* sp.	CPCC 278	12	8.3	0.14 ± 0.00	0.02 ± 0.01
Bacillariophyceae	Bacillariales	*Nitzchia* sp.		13	6.09	0.56 ± 0.02	0.04 ± 0.01
	Tabellariales	*Diatoma tenuis*	CPCC 62	14	6.14	0.45 ± 0.01	0.10 ± 0.02
	Tabellariales	*Tabellaria fenestrata*	CPCC 619	15	5.94	0.50 ± 0.07	0.07 ± 0.04
Dinophyceae (dinoflagellates)	Peridianales	*Peridinium cinctum*	SCCAP K-1721	16	32.29	0.13 ± 0.00	0.04 ± 0.00

**Table 2:** Pseudo-F and Monte Carlo p-values (P(MC) for PERMANOVA analysis of ω-3 and ω-6 PUFA of phytoplankton strains by the phytoplankton group and phase and mix of them as factors.

		PERMANOVA			
Unit	Factors	Df	Pseudo-F	exp %	P(MC)
Contribution	Group	5	141.46	84	0.001 *
	Phase	1	7.2967	1	**0.001**
	GroupxPhase	5	7.8303	5	**0.001**
Biomass content	Group	5	39.307	69	**0.001**
	Phase	1	1.6199	1	0.154
	GroupxPhase	5	1.075	2	0.345
Cell content	Group	5	33.402	65	**0.001**
	Phase	1	1.2592	0	0.233
	GroupxPhase	5	1.3961	2	0.12
Production	Group	5	40.176	66	**0.001**
	Phase	1	2.9217	1	0.019
	GroupxPhase	5	3.7874	6	**0.001**

* bold value means statistically significant different.

**Table 3:** Similarity percentages of SIMPER analysis used to assess similarity within phytoplankton class/group by the different units of the ω-3 and ω-6 PUFA abundance and main PUFAs, explaining most of the similarity. *n* = strain number within taxa + number of growth phases.

		SIMPER	
Taxa	Unit	Average Sim. (%)	Main PUFA
Diatom	Contribution	70.7	EPA
(*n* = 3 + 2)	Biomass content	64.8	EPA
	Cell content	60.7	EPA
	Production	72.7	EPA
Golden algae	Contribution	74.6	SDA, ALA, LIN
(*n* = 4 + 2)	Biomass content	65.8	SDA, ALA, LIN
	Cell content	52.3	SDA, ALA, LIN
	Production	60.7	SDA, ALA, LIN
Dinoflagellate	Contribution	86.5	DHA, EPA
(*n* = 1 + 2)	Biomass content	85.8	DHA, EPA
	Cell content	83.1	DHA, EPA
	Production	81.3	DHA, EPA, SDA
Cryptophytes	Contribution	91.3	SDA, ALA, EPA
(*n* = 2 + 2)	Biomass content	88.5	SDA, ALA, EPA
	Cell content	51.8	SDA, ALA, EPA
	Production	83.3	SDA, ALA, EPA
Cyanobacteria	Contribution	70.7	ALA, LIN
(*n* = 2 + 2)	Biomass content	62.3	ALA, LIN
	Cell content	51.8	ALA, LIN
	Production	41.3	ALA, LIN
Green algae	Contribution	82.3	ALA
(*n* = 4 + 2)	Biomass content	57.1	ALA
	Cell content	64.6	ALA
	Production	49.2	ALA

## Supplementary Materials

The following are available online at https://www.mdpi.com/2218-273X/10/4/559/s1, Figure S1: Development of phytoplankton biomass (as mg C L^−1^) in L. Vesijärvi (data from the database of Finnish Environment Institute) and derived PUFA availability per liter. Figure S2: Development of phytoplankton biomass (as mg C L^−1^) in L. Vesijärvi (data from the database of Finnish Environment Institute) and the derived PUFA availability per phytoplankton carbon content. Table S1: FA profiles of cultured phytoplankton strains.
